# Unsupervised Classification of Polarimetric SAR Image Based on Geodesic Distance and Non-Gaussian Distribution Feature

**DOI:** 10.3390/s21041317

**Published:** 2021-02-12

**Authors:** Junrong Qu, Xiaolan Qiu, Chibiao Ding, Bin Lei

**Affiliations:** 1 Aerospace Information Research Institute, Chinese Academy of Sciences, Beijing 100094, China; qujunrong18@mails.ucas.ac.cn (J.Q.); cbding@mail.ie.ac.cn (C.D.); leibin@mail.ie.ac.cn (B.L.); 2 Key Laboratory of Technology in Geo-Spatial Information Processing and Application System, Chinese Academy of Sciences, Beijing 100094, China; 3 School of Electronic, Electrical and Communication Engineering, University of Chinese Academy of Sciences, Beijing 101408, China

**Keywords:** classification, geodesic distance, K-Wishart classifier, polarimetric SAR

## Abstract

Polarimetric synthetic aperture radar (PolSAR) image classification plays a significant role in PolSAR image interpretation. This letter presents a novel unsupervised classification method for PolSAR images based on the geodesic distance and K-Wishart distribution. The geodesic distance is obtained between the Kennaugh matrices of the observed target and canonical targets, and it is further utilized to define scattering similarity. According to the maximum scattering similarity, initial segmentation is produced, and the image is divided into three main categories: surface scattering, double-bounce scattering, and random volume scattering. Then, using the shape parameter α of K-distribution, each scattering category is further divided into three sub-categories with different degrees of heterogeneity. Finally, the K-Wishart maximum likelihood classifier is applied iteratively to update the results and improve the classification accuracy. Experiments are carried out on three real PolSAR images, including L-band AIRSAR, L-band ESAR, and C-band GaoFen-3 datasets, containing different resolutions and various terrain types. Compared with four other classic and recently developed methods, the final classification results demonstrate the effectiveness and superiority of the proposed method.

## 1. Introduction

Polarimetric synthetic aperture radar (PolSAR) image classification is an important application in SAR image interpretation. PolSAR obtains scattering information through four channels: HH, HV, VH, and VV (HV means the polarization channel of a horizontal incident and a vertical scattered field, and the other three channels are defined in the same way). As a result, it possesses an advantage in capturing comprehensive information about target scattering characteristics, which may lead to a better interpretation and analysis of PolSAR images.

There are three main categories of PolSAR image classification methods. One is based on the statistical characteristics of PolSAR data [1,2,3,4]. Earlier methods are based on Gaussian distribution, like the classic Wishart maximum likelihood (ML) classifier put forward by Kong et al. [2] and Lee et al. [3]. With the spatial resolution of PolSAR images getting higher, the Wishart classifier no longer fits; as a result, some other non-Gaussian distribution models like K-, G-, and U- distribution are proposed. While U- and G-distribution models have complex parameter estimation and are difficult to be applied, K-distribution is widely used [3].

Another approach is based on intrinsic physical scattering characteristics obtained by polarimetric target decomposition (TD) [5,6,7,8,9]. The idea was first proposed by Huynen, and after that, many decomposition theorems were put forward, like the classic Freeman–Durden decomposition (FDD), Yamaguchi decomposition, and Cloude–Pottier decomposition (CPD).

Consequently, methods combining statistical and physical scattering characteristics were proposed and have been widely used [10,11,12,13,14,15,16]. The scattering entropy (H) and alpha angle (α), two parameters derived from CPD, combining the Wishart classifier (H/α-Wishart) method [10] and the FDD combining the Wishart classifier (FDD-Wishart) method [12] are two well-known methods. Furthermore, many other methods have been developed recently [14,15,16]. For example, Zhao et al. proposed the FDD combining scattering entropy (FDD-H) method [14], Wang et al. proposed the scattering power entropy combining co-polarized ratio method [15], Zhou et al. proposed the hetero polarization ratio-based method [16], etc. However, methods based on FDD may result in negative power; and the polarization information may not be fully utilized in some cases, which may cause misclassification in certain areas. Accordingly, many methods are proposed for better scattering mechanism recognition, like six- and seven-component decomposition [7,17], but most of these have a heavy calculation burden and lack exact physical meaning, thus having a limited application.

Recently, Ratha et al. used the geodesic distance (GD) between Kennaugh matrices for scattering mechanism recognition [18] and further put forward an unsupervised classification scheme (GD-Wishart) following the FDD-Wishart framework [13]. The experimental results in [13] show the GD-based method could circumvent the volume scattering overestimation and the negative power occurrence in the FDD-Wishart method, thus having better scattering mechanisms preservation. Moreover, it has a higher calculation efficiency, whereas it is found that the Wishart classifier may not work well in heterogeneous areas [19].

Given the analysis above, we propose an algorithm utilizing the GD and K-Wishart distribution. A scattering similarity measure derived from GD is introduced for a better scattering mechanism division, and K-Wishart distribution is adopted to improve the performance in heterogeneous areas. Experiments on three real PolSAR datasets are conducted to evaluate the effectiveness and robustness of our method.

## 2. Methodology

### 2.1. Geodesic Distance and Scattering Similarity Measure

Scattering (or Sinclair) matrix ***S*** is applied to characterize scattering properties of PolSAR targets. It can be defined as

(1) $$S=\left[\begin{array}{ll} S_{hh} & S_{hv}\\ S_{vh} & S_{vv} \end{array}\right]$$

***S*** is a complex matrix. $S_{vh}$ represents the scattering coefficient, with v and h denoting that the incident and scattered field polarization are vertical and horizontal, respectively. If ***S*** satisfies reciprocity theorems, $S_{vh}=S_{hv}$.

Scattering from terrain targets is generally complicated, and analyses are usually conducted on several basic scatterers. In this paper, we use the geodesic distance and compare target scattering with three canonical scattering mechanisms: odd-scattering, even-scattering, and volume-scattering.

The 4 × 4 Kennaugh matrix ***K*** is obtained by:

(2) $$K=\frac{1}{2}\times (A^{*}\left(S⊗S^{*}\right)A^{-1})$$

where $A=\left[\begin{array}{cccc} 1 & 0 & 0 & 1\\ 1 & 0 & 0 & -1\\ 0 & 1 & 1 & 0\\ 0 & j & -j & 0 \end{array}\right]$, ⊗ represents tensor Kronecker product of matrix, $j=\sqrt{-1}$.

***K*** is a real matrix, retaining scattering information with a low calculation burden. The ***K*** matrix of three elementary scatterers are:

(3) $$K_{a}=\left[\begin{array}{cccc} 1 & 0 & 0 & 0\\ 0 & 1 & 0 & 0\\ 0 & 0 & 1 & 0\\ 0 & 0 & 0 & -1 \end{array}\right]\;K_{b}=\left[\begin{array}{cccc} 1 & 0 & 0 & 0\\ 0 & 1 & 0 & 0\\ 0 & 0 & -1 & 0\\ 0 & 0 & 0 & 1 \end{array}\right]\;K_{c}=\left[\begin{array}{cccc} 1 & 0 & 0 & 0\\ 0 & 1/2 & 0 & 0\\ 0 & 0 & 1/2 & 0\\ 0 & 0 & 0 & 0 \end{array}\right]$$

where subscript a denotes odd-scattering, b denotes even-scattering, and c denotes volume-scattering, modeled by trihedral, dihedral, and random volume scatterers, respectively.

Ratha [13,19] proposed to use the *GD* to acquire a similarity between ***K*** matrices. *GD* is proposed based on the shortest distance of two points projected on the unit sphere of arbitrary dimension. The *GD* between two ***K*** matrices can be defined as:

(4) $$GD\left(K_{1},K_{2}\right)=\frac{2}{\pi }\cos^{-1}\frac{\mathrm{Tr}\left(K_{1}^{T}K_{2}\right)}{\sqrt{\mathrm{Tr}\left(K_{1}^{T}K_{1}\right)}\sqrt{\mathrm{Tr}\left(K_{2}^{T}K_{2}\right)}}$$

where superscript T is the transpose operator, Tr is the trace operator, and 2/π is used to transform *GD* into normalized radian data.

*GD* has a meaningful property that remains invariant in the case of scaling, that is $GD\left(\lambda_{1}K_{1},\lambda_{2}K_{2}\right)=GD\left(K_{1},K_{2}\right)$. This implies that *GD* is only related to the scattering mechanism, and scattering power fluctuation does not affect target interpretation.

As *GD* represents the distance between ***K***-matrix projections, the complementary, similarity measure can be put as (1−GD). The scattering similarity measure can be obtained by (5):

(5) $$f_{i}=\left[1-\mathrm{GD}\left(K,K_{i}\right)\right],0\leq \gamma_{i}=f_{i}/\sum_{i}f_{i}\leq 1$$

In (5), $\gamma_{i}$ is normalized, and i∈{a,b,c} corresponds to three specific scattering types.

Initial scattering-type segmentation is realized by dividing pixels into the category with maximal similarity.

### 2.2. K-Wishart Classifier

Regions of low-resolution images or homogeneous high-resolution images have invariant radar cross section (RCS); thus, complex Wishart distribution [10] is suitable for their description, whereas in heterogeneous regions of high-resolution data, the RCS fluctuates. As a result, the regions have non-Gaussian statistical properties, and the complex Wishart classifier is not applicable. Lee et al. [20] derived the K-distribution of covariance matrix ***Z***, which can better describe the statistical texture characteristics of less homogeneous areas. Doulgeris et al. [4] put forward that covariance matrix ***Z*** follows a K-Wishart distribution, and the probability density function (PDF) is:

(6) $$P(Z)=\frac{2|Z{|}^{n-q}{\left(n\alpha \right)}^{(\alpha +qn)/2}K_{\alpha -nq}(2\sqrt{\left.n\alpha Tr\left(V^{-1}Z\right)\right)}}{R(n,q)|V{|}^{n}\Gamma (\alpha )Tr{\left(V^{-1}Z\right)}^{-(\alpha -qn)/2}}$$

in which Z is the covariance matrix after the n-look process, $Z=(1/n)\sum_{k=1}^{n}u(k)u{\left(k\right)}^{H}$, u(k) denotes the k-th single-look sample, $u={\left(S_{hh},\sqrt{2}S_{hv},S_{vv}\right)}^{T}$, q denotes the dimension of u, $R(n,q)=\pi^{q/2(q-1)}\Gamma (n)\cdots \Gamma (n-q+1)$, $K_{n}(\cdot )$ is the n-th order modified Bessel function of the second kind. α is the shape parameter, and the higher its value, the smoother the image.

Utilizing Bayesian maximum likelihood (ML) classifier, logarithm (6) and taking the opposite value, we define the K-Wishart distance d from ***Z*** to the m-th cluster center $V_{m}$:

(7) $$\begin{array}{ll} d\left(Z,V_{m}\right) & =n\ln\left|V_{m}\right|+\ln[\Gamma (\alpha )]+\ln R(n,q)-\ln P(m)\\ & -\frac{\alpha -qn}{2}\ln Tr\left(V_{m}^{-1}Z\right)-\frac{\alpha +qn}{2}\ln(n\alpha )\\ & -(n-q)\ln(2|Z|)-\ln K_{\alpha -qn}[2\sqrt{n\alpha Tr\left(V_{m}^{-1}Z\right)}] \end{array}$$

Under the hypothesis that prior probabilities are equal to every class and removing terms not related to clustering, (7) can be simplified as:

(8) $$\begin{array}{ll} d(Z,V_{m}) & =\ln(\Gamma (\alpha ))-\frac{\alpha +qn}{2}\ln(n\alpha )-\frac{\alpha -qn}{2}\ln\mathrm{Tr}\left(V_{m}^{-1}Z\right)\\ & \;+n\ln\left|V_{m}\right|-\ln K_{\alpha -nq}(2\sqrt{n\alpha Tr\left(V_{m}^{-1}Z\right)}) \end{array}$$

when $d\left(Z,V_{m}\right)⩽d\left(Z,V_{j}\right)j\neq m$, the pixel is classified into the ***m***-th class.

Anthony et al. found the shape parameter α of K-Wishart distribution can characterize PolSAR data distribution [20]. According to statistical results in [20], a larger value of α(α>15)  corresponds to more homogeneous areas, like farmland and sea surface; a lower value of α(α<2) corresponds to highly non-Gaussian distributed areas, like urban areas and boundaries; and a moderate value of α corresponds to non-Gaussian distributed areas. Therefore, using shape parameter α, we can determine the data distribution of different areas.

We use the moment estimation method [4] to estimate the shape parameter α. We calculate relative kurtosis (*RK*), then *RK* is substituted to calculate α, where I is the intensity image.

(9) $$RK=E\left\{I^{2}\right\}/{\left(E\{I\}\right)}^{2},\alpha =1/(RK-1)$$

Gamma distribution can well describe RCS fluctuations of inhomogeneous regions in high-resolution SAR images. Accordingly, the K-Wishart classifier can represent heterogeneous districts well. Experimental results in [4] show that in non-Gaussian distributed areas, the K-Wishart classifier has better performance than the Wishart classifier, while in Gaussian distributed areas, they produce similar results.

### 2.3. Unsupervised Classification

A reasonable scattering mechanism characterization and a suitable classifier can lead to a more desirable classification performance. Based on the above introduction, we propose an unsupervised PolSAR image classification method combining the *GD* and K-distribution. The whole procedure is summarized as follows.

(1) Preprocessing. Filter the original PolSAR data to reduce speckle noise.
(2) Scattering mechanism initialization. Use three elementary scatterers: trihedral, dihedral, and random volume scatterer. Calculate the geodesic distance (*GD*) of each pixel to the three typical scatterers using (4) and derive the scattering similarity measure using (5). According to the maximum similarity to a target, pixels are divided into three scattering categories: odd-scattering, even-scattering, and volume-scattering.
(3) Calculate the shape parameter α of per-pixel using (9).
(4) Further segmentation. Select proper thresholds $\alpha_{1}=2$, $\alpha_{2}=15$ for α and divide each category in (2) into three. If $\;\alpha \leq \alpha_{1}$, classify the corresponding pixels into the category with highly non-Gaussian distribution; if $\alpha_{1}<\alpha <\alpha_{2}$, classify the pixels into the category with non-Gaussian distribution; and if $\alpha \geq \alpha_{2}$, classify the pixels into the category with Gaussian distribution. In this way, each scattering category is further divided into three sub-categories depending on the degree of conformity to Gaussian distribution.
(5) Iteratively meliorate the results by applying a suitable classifier. Perform K-Wishart classifier or Wishart classifier selectively and iteratively to all pixels. Choose Wishart classifier when $\alpha >50\left(\mathrm{nq}+1\right)/\left(q+1\right)$, and choose the K-Wishart classifier otherwise. Note that all pixels are not allowed to be reclassified to other scattering mechanisms during iteration.
(6) Result output. Set the color table for each major category and corresponding sub-categories. Output the result in (5) according to color settings.

This algorithm fuses the superiority of GD and K-distribution. It is capable of achieving a better scattering mechanism distinction as well as a better classification result.

## 3. Results and Discussion

**Figure 1:**
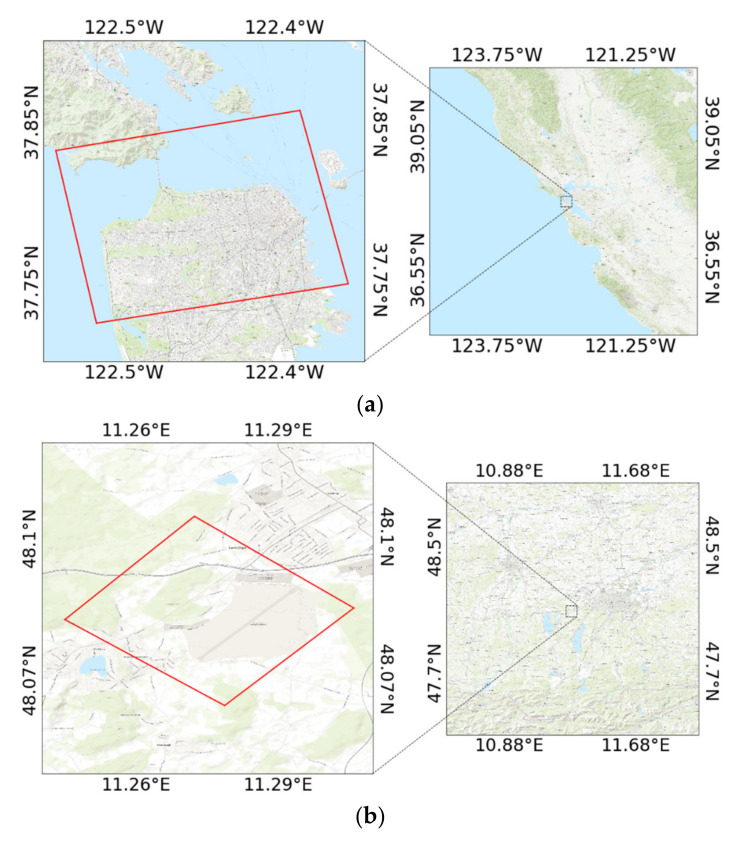

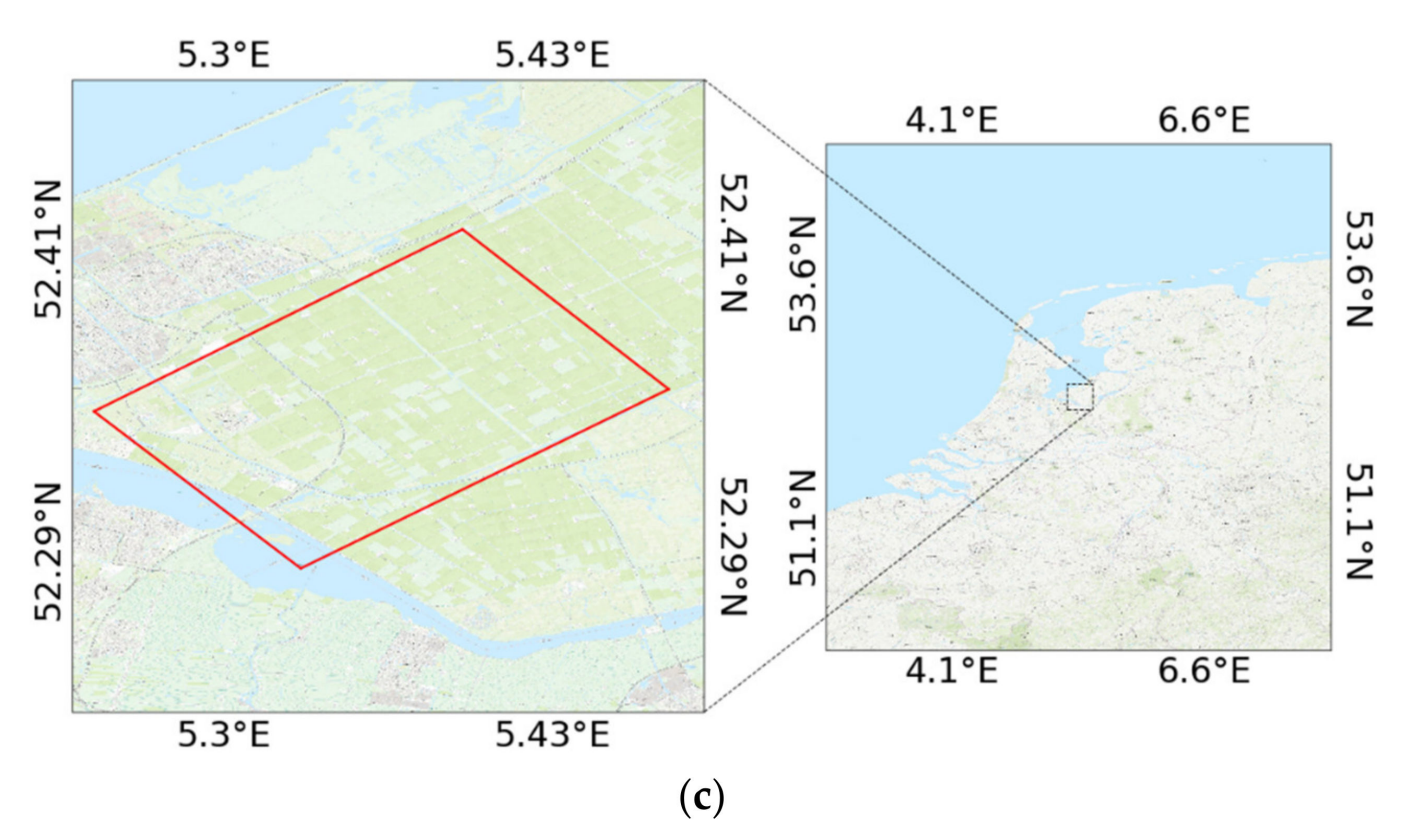
Geographic extents of the three study areas. (**a**) Gaofen-3 data over San Francisco Bay Area, (**b**) ESAR data over Oberpfaffenhofen in Southern Germany, (**c**) AirSAR data over Flevoland in the Netherlands.

Three real PolSAR images acquired by different sensors (Gaofen-3, ESAR, and AIRSAR) are used for experiments. Their locations are shown in Figure 1. Various terrain types are included, ranging from roads, buildings, forests, agricultural areas, to water, etc. To demonstrate the effectiveness of the proposed method, we conduct four different methods for comparison: the classic FDD-Wishart method [12], the FDD combined with scattering entropy (FDD-H) method [14], the recently developed hetero polarization ratio-based ($R_{d}$-based) method [16], and the GD-Wishart method [13].

All datasets were preprocessed by the Refined Lee filter with a 5 × 5 window size and considered the surrounding eight neighborhoods when calculating the shape parameter α.

### 3.1. Experiment Results of Gaofen-3

**Figure 2:**
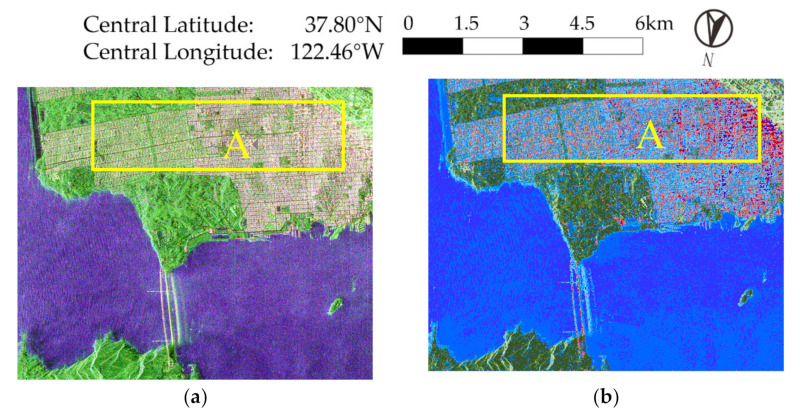

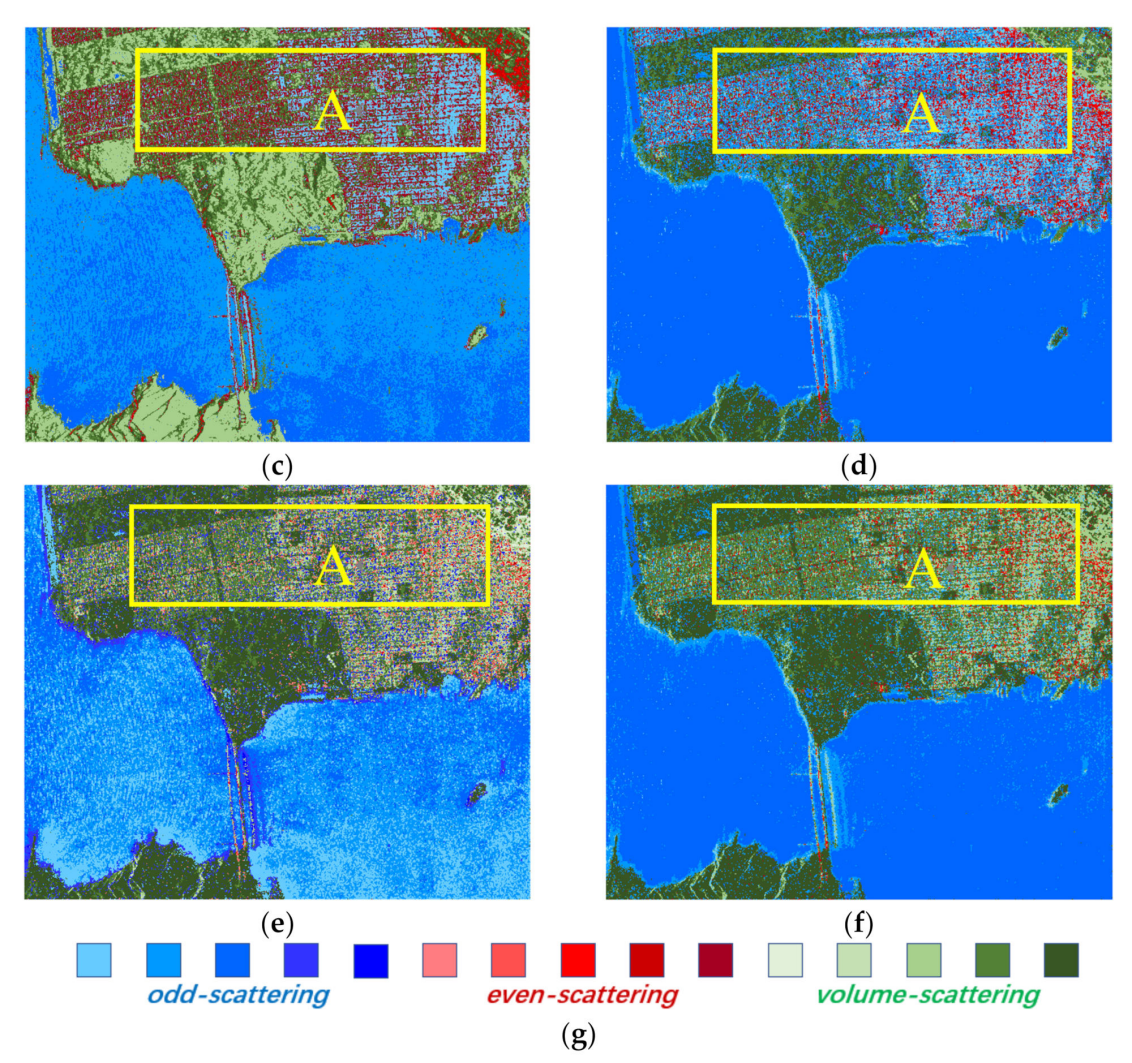
Experiment results of Gaofen-3 data. Zone A in rectangles is enlarged and shown in Figure 3. (**a**) Pauli decomposition, (**b**) Freeman–Durden decomposition (FDD)-Wishart, (**c**) $R_{d}$-based, (**d**) FDD-H, (**e**) geodesic distance (GD)-Wishart, (**f**) proposed method, (**g**) color set for (**b**,**d**–**f**).

The C-band data was acquired over the San Francisco Bay Area by the Chinese Gaofen-3 satellite. The acquisition time was 15 September 2017, and the size of the research area was 1400 × 1700 pixels. Original data were single-looked with azimuth and range resolution of 2.25 m and 5.36 m, respectively. The results are shown in Figure 2.

**Figure 3:**
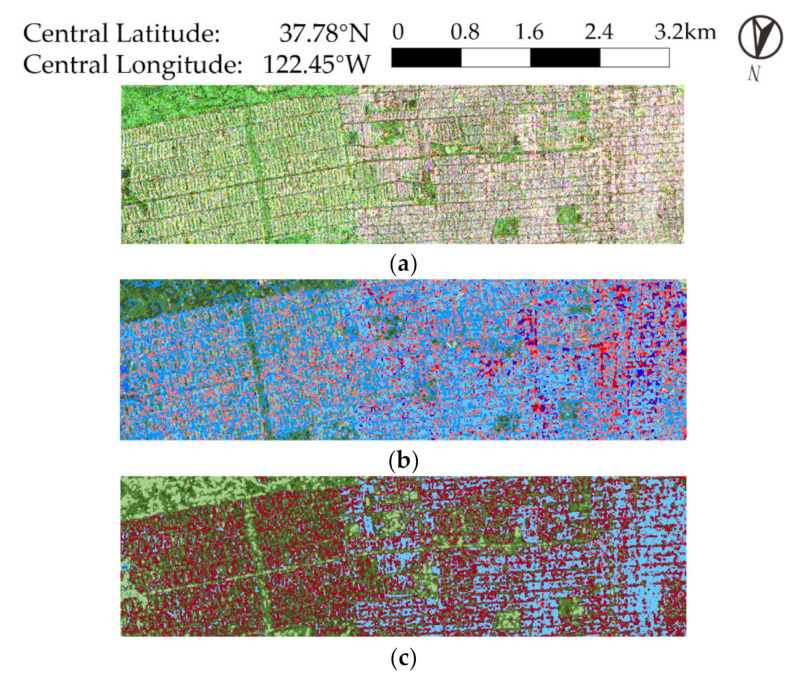

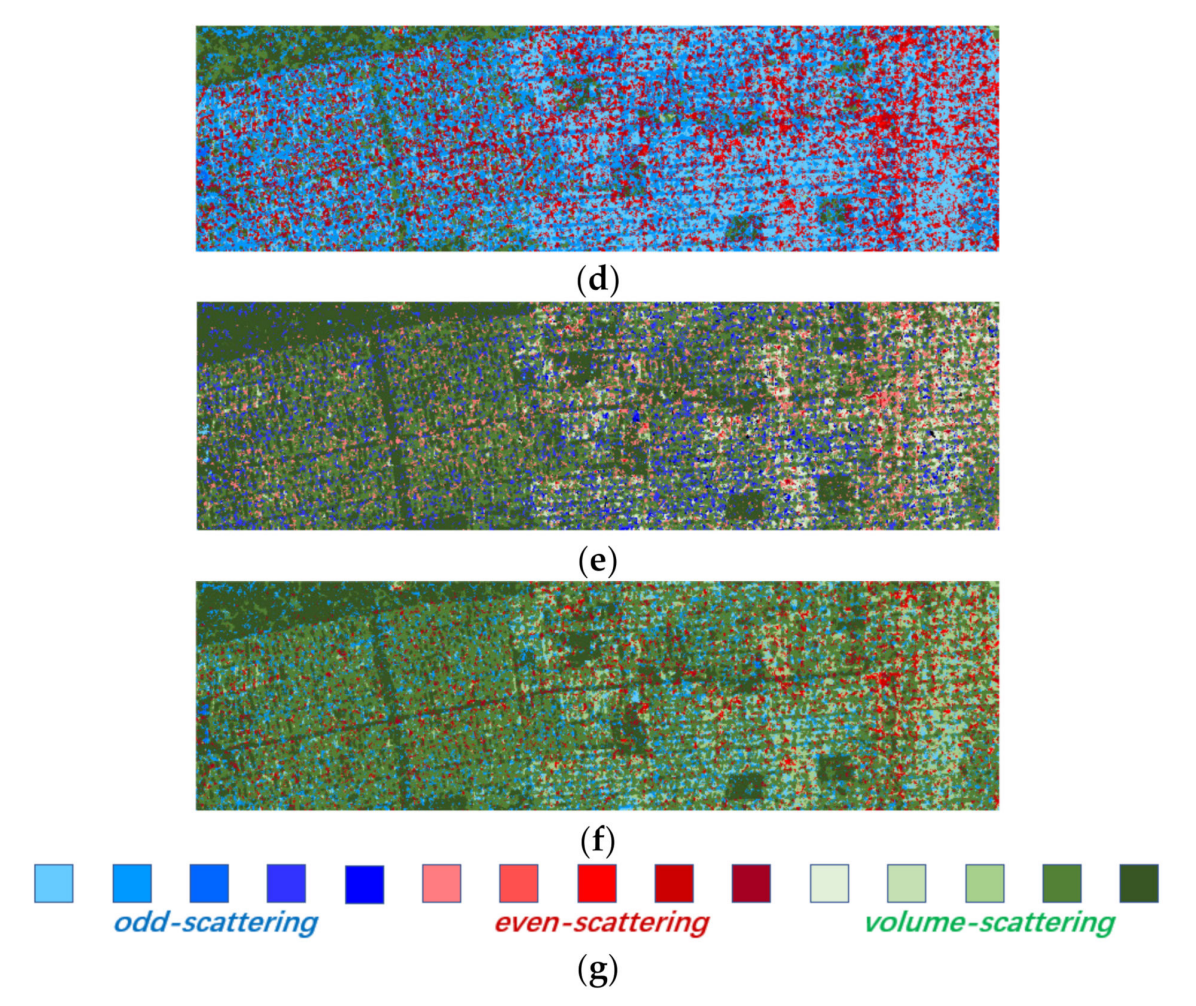
Results of Zone A. (**a**) Pauli decomposition, (**b**) FDD-Wishart, (**c**) $R_{d}$-based, (**d**) FDD-H, (**e**) GD-Wishart, (**f**) proposed method, (**g**) color set for (**b**,**d**–**f**).

We observe that in Figure 2b,d, in Zone A (which is enlarged and displayed in Figure 3), a large portion of vegetation areas is regarded as surface scattering. This phenomenon does not exist in Figure 2e,f, showing that using GD can circumvent volume scattering misclassification and have better scattering mechanism recognition than FDD.

Comparing Figure 2c,e,f, the three methods all have good performance on landcover classification. Roads, playgrounds, and different built-up areas are divided meticulously and clearly, with consistent features within a region and clear boundaries to one another. However, the $\;R_{d}$-based method cannot identify different scattering mechanisms. In addition, we can observe over-segmentation over the ocean in Figure 2c,e. Part of the explanation is that the GD-Wishart and $R_{d}$-based methods rely on span for initialization, but over the sea surface, the signal to noise ratio (SNR) is relatively low and leads to fluctuation of span. In contrast, the proposed method classifies the ocean area almost into one category and distinguishes it clearly from the land.

In summary, the proposed method can achieve a better result both in scattering mechanism preservation and in landcover classification.

### 3.2. Experiment Results of ESAR

The second experiment used L-band data of Oberpfaffenhofen in Southern Germany acquired by the airborne platform ESAR. The original data were single-look and had a high spatial resolution of 3 m in both azimuth and range. The data have a larger size of 2816 × 1540 pixels and had diverse terrain types such as roads, fields, woodlands, airports. Therefore, it may be more difficult in interpretation. Since there is no ground truth, the performance is evaluated on visual qualitative analysis.

**Figure 4:**
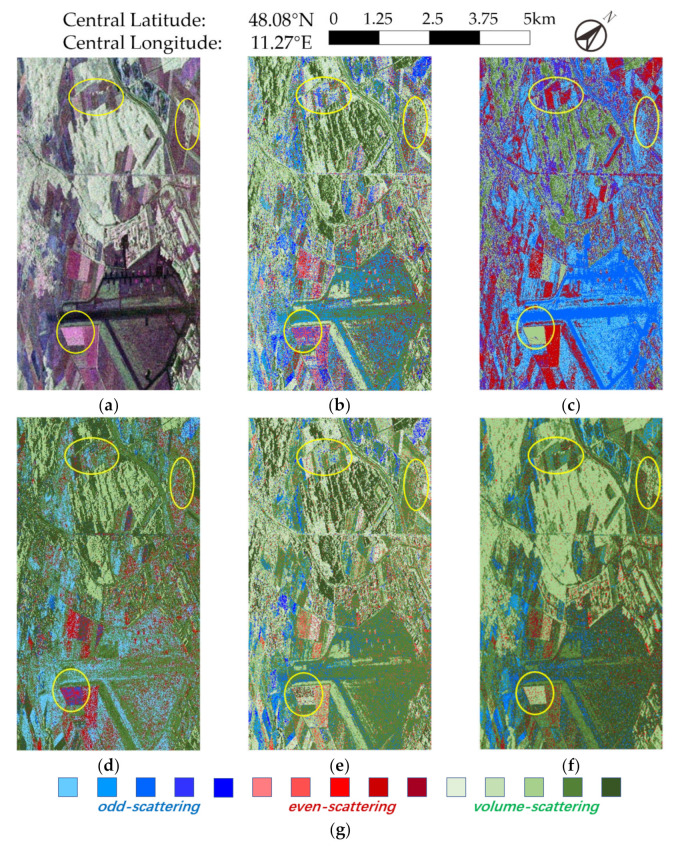
Experiment results of ESAR data. Areas marked by ellipses are selected for specific analysis. (**a**) Pauli decomposition, (**b**) FDD-Wishart, (**c**) $R_{d}$-based, (**d**) FDD-H, (**e**) GD-Wishart, (**f**) proposed method, (**g**) color set for (**b**,**d**–**f**).

We can find that in Figure 4b–e, the edges of different blocks are not clear enough. The interior of the area is not uniform, and there are always small, blocked areas, whether in woodlands or urban areas. Moreover, many pixels are misclassified or over-segmented, e.g., the FDD-based methods Figure 4b,d seriously misclassified vegetation area as surface scattering (the airport in the lower part of the image has more areas appearing blue).

Taking some specific areas in ellipses for further analysis, in the lower-left elliptic area, we see severe clutter and extreme unevenness in Figure 4b,d,e. In addition, there are almost no clear boundaries with the surrounding areas. A similar phenomenon can also be seen in the upper two marked areas in Figure 4b–e, though Figure 4c performs well in the lower left circle area. The corresponding result in Figure 4f is smooth inside and has clear edges, and can be clearly distinguished from surrounding districts. Furthermore, the proposed method can maintain borders and roads well without manifest noise and misclassification. For districts such as airport apron (which is homogeneous), the proposed method does not show an obvious advantage and has similar results to Figure 4e.

In this way, the proposed method can preserve uniformity within a region and represent boundaries, thus distinguishing different regions clearly, and has better classification performance for this dataset.

### 3.3. Experiment Results of AIRSAR

The third research area was located in Flevoland in the Netherlands. The data were acquired by the airborne platform AIRSAR on 16 August 1989. The original 4-look L-band data had a size of 750 × 1024 with a spatial resolution of 6.6m and 12.1 m in range and azimuth, respectively. Results with different methods are shown below.

**Figure 5:**
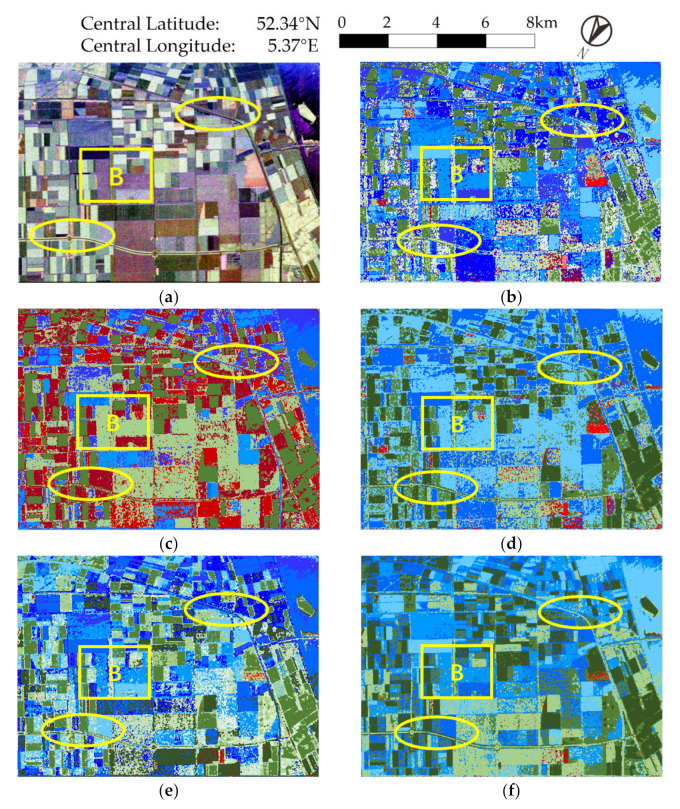

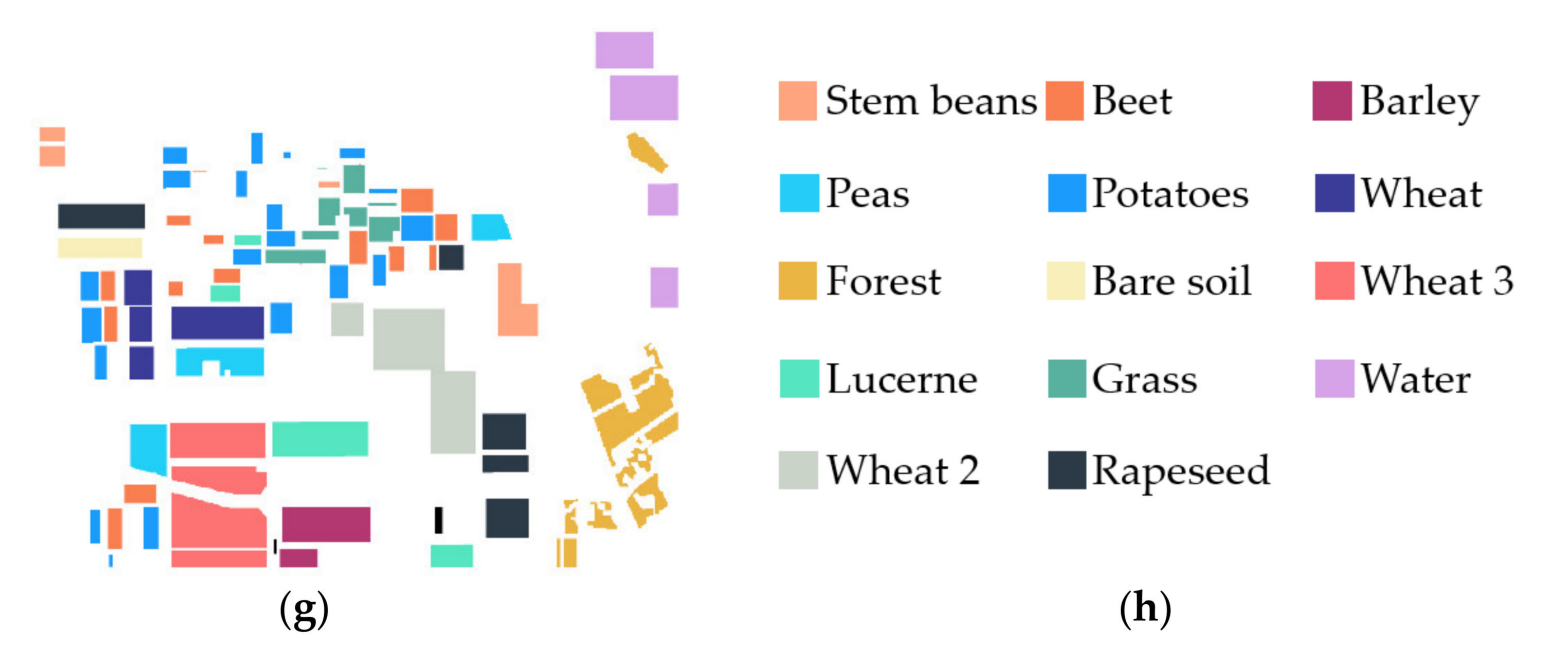
Experiment results of AIRSAR data. Areas in ellipses are taken as a comparison example. Zone B in rectangles is selected for further analysis in Figure 6 and Table 1. (**a**) Pauli decomposition, (**b**) FDD-Wishart, (**c**) $R_{d}$-based, (**d**) FDD-H, (**e**) GD-Wishart, (**f**) proposed method, (**g**) ground truth, (**h**) ground truth legend.

Figure 5b–e shows contrasting experiment results. There are many small holes and it is not smooth enough in each parcel, so the result is undesirable. The outcome in Figure 5f shows that besides utilizing GD for better scattering mechanisms maintenance, the proposed method also has better preservation of ground edges and details. Moreover, we can see from the areas in ellipses that the algorithm can distinguish the road from surrounding farmland clearly, and the division of cropland is more accurate.

**Figure 6:**
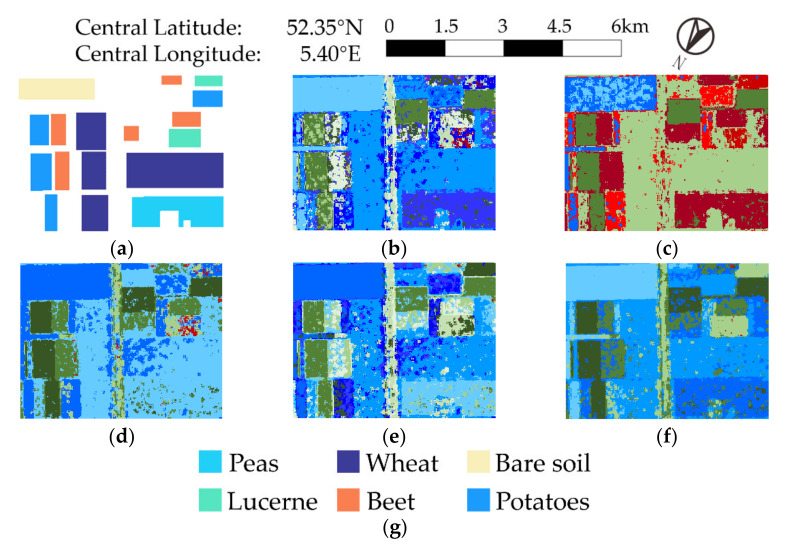
Results of Zone B. (**a**) Ground truth, (**b**) FDD-Wishart, (**c**) $R_{d}$-based, (**d**) FDD-H, (**e**) GD-Wishart, (**f**) proposed method, (**g**) ground truth legend.

We selected an area of interest (Zone B) to evaluate the performance qualitatively. Zone B in Figure 5 is magnified, and the details are displayed and compared. The result is shown in Figure 6, and classification accuracy is shown in Table 1.

Comparing results in Figure 6, the result obtained by the proposed method of Figure 6f has better intra-area uniformity and higher inter-area contrast. Table 1 shows that the overall classification accuracy using the proposed method is 89.48%, which is 6.38% higher than the second FDD-Wishart algorithm and 11.64% higher than the 77.84% of the GD-Wishart algorithm. In addition, the proposed method obtains a comparatively ideal result for each category.

In summary, the results suggest the superiority of the proposed method, whether in visual effect or in classification accuracy.

## 4. Conclusions

A novel unsupervised PolSAR classification algorithm combining the geodesic distance and the K-Wishart ML classifier is presented in this paper. Considering that a better scattering mechanism division can be achieved by scattering similarity derived from the geodesic distance between Kennaugh matrices, and a better description in heterogeneous areas can be achieved by the K-Wishart classifier, we use the scattering similarity to canonical scatterers to obtain initial segmentation, and then use the shape parameter α of K-distribution to further divide each scattering mechanism into three sub-categories. Finally, an iterative algorithm is used to improve the result. Experiments conducted on three real datasets demonstrate the effectiveness of the algorithm and the improvement of classification performance. Moreover, our algorithm divides an image into fixed numbers of categories and avoids clustering and merging, which makes the algorithm concise and easy to implement.

Future work may consider finding an adaptive method to identify the proper number of classes, and adding appropriate feature parameters during initialization according to the complexity of the image.

## Figures and Tables

**Table 1:** Overall Classification Accuracy of Zone B by Different Methods.

	Method	FDD-Wishart	R_d_-Based	FDD-H	GD-Wishart	Proposed
Accuracy						
Peas		88.17	0	0	83.60	84.36
Wheat		92.85	98.87	84.76	80.97	91.61
Bare soil		99.85	50.91	99.96	99.92	99.96
Lucerne		41.94	66.83	42.29	57.84	98.55
Beet		56.85	96.93	85.43	30.68	66.82
Potatoes		75.07	96.74	97.61	92.79	96.13
Overall		83.01	75.97	72.62	77.84	89.48

## Data Availability

Data sharing is not applicable to the first dataset in this study. And the original data of the last two datasets can be found in the link: https://earth.esa.int/web/polsarpro/data-sources/sample-datasets.
